# Transcriptional Profiling of Somatostatin Interneurons in the Spinal Dorsal Horn

**DOI:** 10.1038/s41598-018-25110-7

**Published:** 2018-05-01

**Authors:** Alexander Chamessian, Michael Young, Yawar Qadri, Temugin Berta, Ru-Rong Ji, Thomas Van de Ven

**Affiliations:** 1 Department of Anesthesiology, Duke University Medical Center, Durham, North Carolina 27710 USA; 2 Department of Neurobiology, Duke University Medical Center, Durham, North Carolina 27710 USA; 3 Medical Scientist Training Program, Duke University School of Medicine, Durham, North Carolina 27710 USA; 4 Department of Pharmacology and Cancer Biology, Duke University Medical Center, Durham, North Carolina 27710 USA; 5 Pain Research Center, Department of Anesthesiology, University of Cincinnati Medical Center, Cincinnati, Ohio, 45267 USA

## Abstract

The spinal dorsal horn (SDH) is comprised of distinct neuronal populations that process different somatosensory modalities. Somatostatin (SST)-expressing interneurons in the SDH have been implicated specifically in mediating mechanical pain. Identifying the transcriptomic profile of SST neurons could elucidate the unique genetic features of this population and enable selective analgesic targeting. To that end, we combined the Isolation of Nuclei Tagged in Specific Cell Types (INTACT) method and Fluorescence Activated Nuclei Sorting (FANS) to capture tagged SST nuclei in the SDH of adult male mice. Using RNA-sequencing (RNA-seq), we uncovered more than 13,000 genes. Differential gene expression analysis revealed more than 900 genes with at least 2-fold enrichment. In addition to many known dorsal horn genes, we identified and validated several novel transcripts from pharmacologically tractable functional classes: Carbonic Anhydrase 12 (*Car12*), Phosphodiesterase 11 A (*Pde11a*), and Protease-Activated Receptor 3 (*F2rl2*). *In situ* hybridization of these novel genes showed differential expression patterns in the SDH, demonstrating the presence of transcriptionally distinct subpopulations within the SST population. Overall, our findings provide new insights into the gene repertoire of SST dorsal horn neurons and reveal several novel targets for pharmacological modulation of this pain-mediating population and treatment of pathological pain.

## Introduction

Mechanical pain is one of the chief symptoms in many pathological pain conditions^1^. Accordingly, understanding the spinal circuits underlying this component of pain perception has been a central aim of preclinical pain research^2,3^. Recent studies employing genetic tools to manipulate specific spinal circuits have greatly expanded our understanding of this question^4–7^. In this way, it was discovered that a population of Somatostatin-expressing (SST) excitatory interneurons in the superficial dorsal horn is required for mechanical pain, as demonstrated by the complete absence of mechanical pain when SST neurons were ablated^6^. It appears that other sensory modalities such as thermosensation and innocuous touch were left undisturbed, indicating that SST interneurons play a specific and restricted role. With the functional role of SST neurons now well-established, it would be advantageous to comprehensively characterize the repertoire of genes expressed by SST neurons, thus providing a basis for their unique properties and highlighting potential targets for selective pharmacological manipulation.

RNA-sequencing (RNA-seq) is a powerful tool to uncover the transcriptome of cells and tissues. To date, gene expression studies of the dorsal horn have used RNA isolated from bulk spinal tissue, which represents a mixture of genes expressed by multiple neuronal and non-neuronal cell types^8,9^. To examine the transcriptional profile of a single neuronal population, isolating RNA solely from those cells is necessary^10^.

To access the transcriptome of specific cell types, various methods have been developed. Dissociation of intact neurons from neural tissue coupled with fluorescence activated cell sorting (FACS) has been used to profile neurons and glia in the CNS, but this method requires relatively harsh protease treatments at warm temperatures, which induces artifactual gene expression signatures due to processing^11–13^.

To obviate the need to dissociate neural cells, a cell type-specific method called Isolation of Tagged Nuclei from Specific Cell Types (INTACT) was developed^14,15^. In this method, nuclei conditionally express a fusion protein comprised of green fluorescent protein (GFP) and the native nuclear membrane protein SUN1^16^. Tagged nuclei can then be captured either by immunoprecipitation or fluorescence activated nuclear sorting (FANS) for downstream genomic analysis. The INTACT method has many benefits. Nuclei are readily obtained from fresh or frozen tissue at cold temperatures by simple mechanical homogenization, which eliminates the concern of processing artifacts and offers the unique possibility to use post-mortem human samples^17,18^. Because intercellular connections are destroyed by mechanical dissociation, biases toward specific cell types due to viability or cytoarchitecture are greatly minimized^13^. Moreover, it has been demonstrated that the transcriptional signature of nuclear RNA is highly concordant with that of whole cells^18,19^.

In this study, we optimized INTACT for use on spinal cord tissue to profile the transcriptome of dorsal horn SST neurons. We determined the expression levels of >13,000 genes, 901 of which were significantly enriched in the SST population compared to all dorsal horn cells. Using *in situ* hybridization and immunohistochemistry, we validated the expression of several novel and highly enriched genes in SST neurons that could make attractive therapeutic targets. Furthermore, we show the SST population is transcriptionally heterogeneous and contains multiple subpopulations.

## Experimental Procedures

### Animals

All the animal procedures were approved by the Institutional Animal Care and Use Committee of Duke University. Animal experiments were conducted in accordance with the NIH Guide for the Care and Use of Laboratory Animals. Mouse strains used included *Sst-IRES-Cre*^20^ (Jax#013044) and *R26-CAG-LSL-Sun1-sfGFP-Myc*^14^ (Jax # 021039). We refer to this line as *Sun1-GFP*^*(fl*/*fl)*^. To generate SST^GFP^ mice, homozygous *Sst-ires-Cre* males were bred with homozygous *Sun1-GFP*^(*fl*/*fl*)^ females to create compound heterozygous offspring, which were used in all subsequent experiments. To generate control animals for comparison to SST^GFP^, we bred homozygous *Sun1*-GFP^*(fl*/*fl*)^ with C57BL6/J mice to obtain offspring with *Sun1-GFP*^*(fl*/+*)*^ genotypes. For some immunohistochemistry of CAR12, we used spinal cord tissue from SST-Tomato mice, which is the product of a cross between homozygotes of the *Sst-ires-Cre* line and Cre-dependent tdTomato reporter line Ai9 (Jax# 007909)^21^. For RNA-seq and microscopy experiments, male mice 8–12 weeks of age were used. Nuclei isolation and Fluorescence Activated Nuclear Sorting (FANS)

A dorsal segment from the L3-L5 region from each SST^GFP^ or Sun1-GFP^(*fl*/+*)*^ mouse was dissected using small spring scissors and snap frozen on dry ice for later processing. On the day of experiment, the tissue segment was placed in 1 mL of Nuclear Extraction Buffer (NEB) supplemented with RNase and Protease Inhibitors (20 mM Tris HCl pH 8, 5 mM MgCl2, 25 mM KCl, 250 mM Sucrose, 40 U/mL RNasin Plus (Promega), 1 tablet/10 mL Protease Cocktail Mini EDTA-Free (Roche), 1 uM DTT (Sigma), 0.3% NP-40 (Pierce)). Dounce Homogenization (10 strokes Pestle A, 10 strokes Pestle B) was performed to liberate the nuclei using a 2 ml homogenizer (Sigma). The homogenate was filtered through a 50 μm Partec filter (Sysmex) into a regular (not low-binding) 1.7 ml microcentrifuge tube (Axygen), since low-binding tubes create loose pellets that are easily displaced. The filter was washed with an additional 700 μl NEB and the homogenate was then centrifuged for 10 mins at 4 °C (500 g) to form a loose pellet. The pellet was resuspended with 500 μl of NEB using a p1000 pipettor with regular-bore tips and 10 aspiration/dispense cycles, and filtered through a 20 μm Partec filter into a 5 ml polypropylene tube. DAPI (4′,6-diamidino-2-phenylindole) was added to the sample to a final concentration of 5 ng/ml. We made several modifications to the INTACT procedure by Mo *et al*.^14^ to simplify the workflow and make the procedure suitable for small spinal cord samples: (1) All volumes and vessels were scaled down to accommodate the smaller size of a dorsal lumbar spinal segment from mouse compared to cortex; (2) Density gradient separation (e.g. Iodixanol) was removed, as we noticed it caused clumping of nuclei and added no additional benefit; (3) FANS, as opposed to bead immunoprecipitation, was used in order to most specifically and cleanly isolate the relatively low number of GFP+ nuclei in the dorsal segment.

FANS was performed using a BD FACSAria II sorter (BD Biosciences) using a pressure of 35 pounds per square inch (psi) and a 100 μm sort nozzle. Gates were established to capture singlet nuclei with high DAPI staining intensity. Side scatter (SSC) and forward scatter (FSC) were first used to isolate singlet nuclei from debris and multiplets. Then DAPI signal was used to separate additional debris from intact nuclei. Nuclei were sorted into collection tubes using ‘purity’ mode to exclude any potential multiplets. For SST^GFP^, 5000 DAPI+/GFP+ events were sorted into 350 μl of RNAaqeous Micro (Life Technologies) lysis buffer. For the total nuclei control sample from *Sun1-GFP*^*(fl*/+*)*^ mice, 5000 DAPI+/GFP− events were isolated in the same manner. FANS data were analyzed using FlowJo 3.0 (FlowJo LLC). For each mouse line, n = 3 samples were isolated. Captured samples were placed on ice and immediately processed for RNA isolation.

### RNA library construction and sequencing

RNA isolation was performed using the RNaqeuous Micro kit (Life Technologies) according to the manufacturer’s instructions. DNase digestion was not performed at this step, since the downstream library preparation included a DNase step. For library preparation, all samples were processed at the same time using the SoLo RNA-Seq kit (NuGen Technologies) according to the manufacturer’s instructions in a PCR-clean laminar flow hood. For the PCR amplification step, 17 cycles were used, as this was the optimal number of cycles determined in a prior qPCR optimization assay according to the manfacturer’s instructions. Library concentration was assessed with the Qubit 2.0 fluorometer and dsDNA HS assay (Life Technologies), checked for quality on the Bioanalyzer (Agilent) and then run on the HiSeq 2500 (Illumina) using a 50 base-pair, single-end read protocol.

### Bioinformatic Analysis

RNA-seq data was processed using the TrimGalore toolkit^20^ which employs Cutadapt to trim low quality bases and Illumina sequencing adapters from the 3′ end of the reads. Only reads that were 20 nt or longer after trimming were kept for further analysis. Reads were mapped to the GRCm38v68 version^22^ of the mouse genome and transcriptome using the STAR RNA-seq alignment tool^23^. Reads were kept for subsequent analysis if they mapped to a single genomic location. Gene counts were compiled using the HTSeq tool. For this analysis, we used the standard method of only counting reads that mapped to known exons. Only genes that had at least 10 reads in any given library were used in subsequent analysis. Normalization and differential expression was carried out using the DESeq2^24^ Bioconductor^25^ package with the R statistical programming environment. The false discovery rate was calculated to control for multiple hypothesis testing.

Heatmap generation for function classes was performed using the pheatmap package (R). Only coding genes with log2FC > 1, q-value < 0.01, coefficient of variation (CV) < 65% and normalized counts > 20 were included. Pathway analysis was performed using the Ingenuity Pathway Analysis program (Qiagen), using only upregulated genes with q-value < 0.01 and log2FC > 1^26^. Gene set enrichment analysis^27^ was performed to identify differentially regulated pathways and gene ontology terms for each of the comparisons performed. Up-regulated genes matching the inclusion criteria above were analyzed using Ingenuity Pathway Analysis (Qiagen).

Gene lists for functional classes were curated from the IUPHAR database^28^, the Pain Genes database^29^, Neuropeptides Database^30^ and Riken Transcription Factor Database^31^.

### *In situ* Hybridization (ISH) and Immunohistochemistry (IHC)

Animals were deeply anesthetized with isoflurane and transcardially perfused with 4% paraformaldehyde in phosphate-buffered saline (pH 7.4). After perfusion, lumbar spinal cord segments (L3-L5) were removed and postfixed in the same fixative for 2 hours at 4 °C. Then, the tissues were cryopreserved in 30% sucrose/PBS solution for at least 24 hours. For immunohistochemistry (IHC), tissues were blocked in blocking/staining buffer consisting of 1 × PBS (Life Technologies), 1% Bovine Serum Albumin (BSA, Cell Signaling Technologies) and 0.4% Triton-X 100 (Bio-rad) for 1 hour at room temperature (RT). After blocking, the sections were then incubated overnight with primary antibodies diluted in blocking buffer at 4 °C. After washing 3 times in PBS for 5 minutes at RT, sections were incubated with the appropriate secondary antibody for 1 hour at RT followed by 3 washes in PBS for 5 minutes. Before mounting, some sections were counterstained with DAPI (Life Technologies). Slides were then mounted in Prolong Gold (Life Technologies). Primary antibodies were used at the following dilutions: chicken anti-GFP (1:1000, Abcam, ab13970), goat anti-mCAR12 (1:500, R&D Systems,AF2345). Secondary antibodies were as follows: Cy3-conjugated anti-Goat Cy3 (1:1000, Jackson ImmunoResearch), Alexa 488-conjugated anti-Chicken (1:1000, Life Technologies).

For *in situ* hybridization, lumbar spinal cord segments were dissected and post-fixed for 2 hours at 4 °C. The spinal cords were cryo-sectioned to 14 um, thaw-mounted onto Superfrost Plus (Fisher Scientific) slides, allowed to dry for 20 minutes at RT, and then stored at −80C. *In situ* hybridization was performed using the RNAscope system (Advanced Cell Diagnostics). Tissue pretreatment consisted of 30 minutes of Protease IV at RT. Following pretreatment, probe hybridization and detection with the Multiplex Fluorescence Kit v2 were performed according to the manufacturer’s protocol. Probes included Mm-Sst-C1 (#404631), Mm-Sst-C2 (#404631-C2), Mm-Pde11a (#481841), Mm-Car12 (#429991), Mm-F2rl2 (#489591), Mm-Gpr26 (#317381), Mm-Nmur2 (#314111), Mm-Grp (#317861). In some cases, *in situ* hybridization was followed by IHC to visualize GFP because the native GFP signal was destroyed by the RNAscope pretreatment. After the RNAscope detection, the tissue sections were processed for IHC as indicated above to reveal GFP fluorescence. Following ISH or combined ISH/IHC, slides were mounted using Prolong Gold.

Fluorescence was detected using an epifluorescence microscope (Nikon Eclipse NiE). Images were taken at 20x and 40x magnification with Z-stacks. Stacked images were combined using the Maximum Intensity Projection function in the Elements software (Nikon). For quantification, all images were taken using the same acquisition settings. Cell counts were performed on the entire dorsal horn for each marker. Three to five sections were selected from 3–4 animals, and the counts from all sections for each animal were averaged. Each first section was selected randomly, and each subsequent section was at least 140 um apart, in order to avoid double-counting. For co-expression quantification, all images in a set underwent the same image post-processing, with a threshold uniformly set on each color channel. Images were processed and analyzed using a custom script written in Matlab (Mathworks). Images were first thresholded manually for each individual channel and the same thresholds were used for all samples in the analysis. Tophat filtering was performed prior to watershed segmentation of the DAPI channel in order to segment individual cells. Cells were determined to be positive for a particular transcript if they contained any pixels above the set threshold within the border defined by the DAPI channel. Images were prepared in Photoshop CC (Adobe).

### Experimental Design and Statistical Analysis

For RNA-seq, samples were prepared from three (n = 3) adult, male mice from the SST^GFP^ line and the *Sun1-GFP*^*(fl*/+*)*^. For differential gene expression analysis, the log2 fold change between GFP + nuclei from SST^GFP^ mice and total DAPI + nuclei from *Sun1-GFP*^*(fl*/+*)*^ was determined using the DESeq2 package in R. Statistical significance was determined using the Wald test statistic. Adjusted p-value, which we refer to the q-value, reflects correction of the p-value for multiple comparisons. We considered a gene to be differentially expressed if the log2FC > 1, q-value < 0.01, coefficient of variation between replicate < 65% and that all replicates in the SST^GFP^ set had at least 20 normalized counts. We used a q-value cutoff that is more stringent than the conventional q < 0.05 in order minimize false positives. For ISH counting, we present the percentage of colocalization events and the standard error of the mean (SEM).

### Data availability

The complete set of raw sequences obtained in this study has been deposited in Sequence Read Archive (SRA) under the study accession no. SRP141141.

## Results

### Isolation of RNA Specifically from Somatostatin Interneurons in the Dorsal Horn

**Figure 1:**
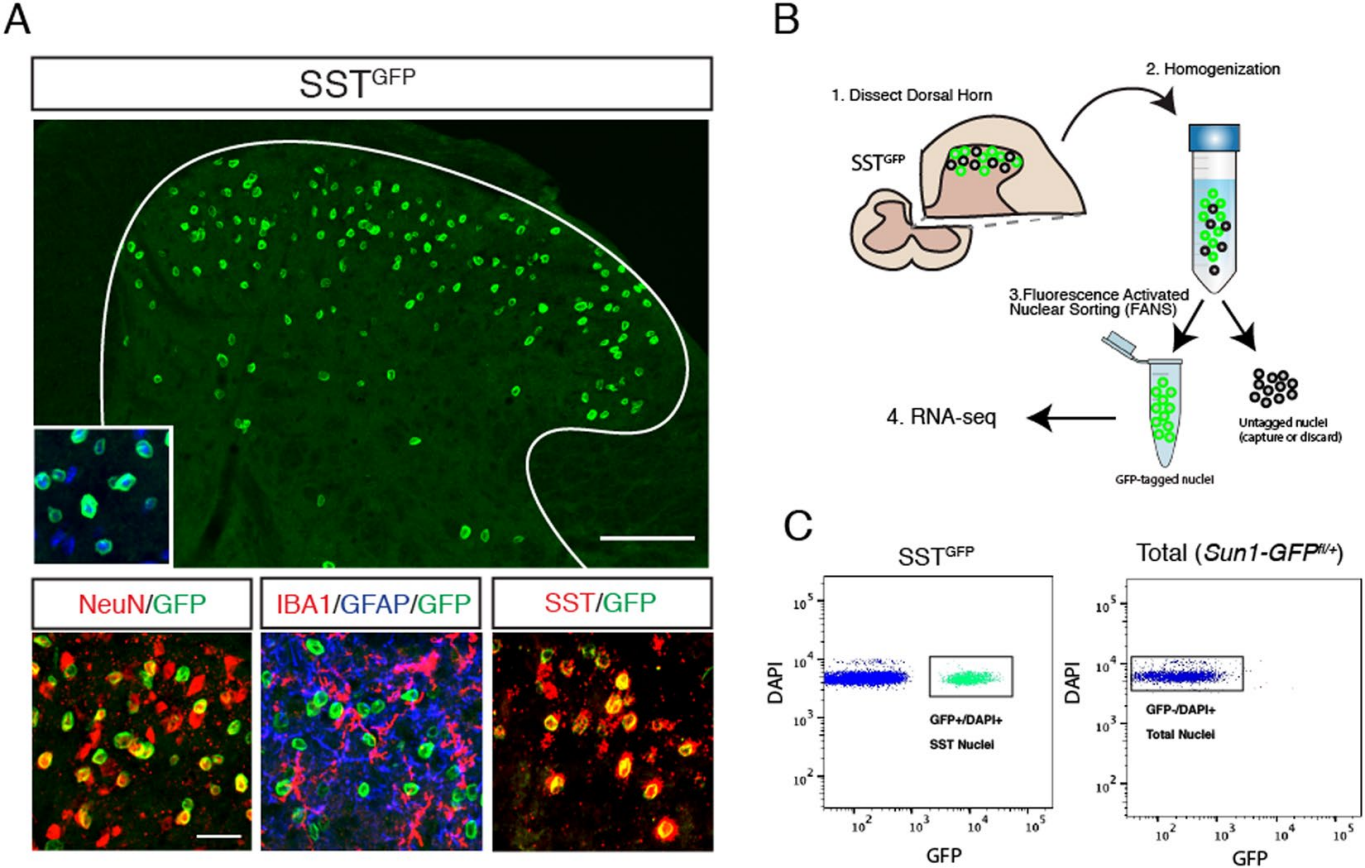
Cell type-specific nuclear tagging and capture of SST neurons in the spinal dorsal horn. (**A**) SST^GFP^ mice express the Sun1-GFP nuclear tag in SST neurons in the lumbar dorsal horn (a). The GFP-tagged SST neurons co-localize with the neuronal marker NeuN (red, b), but not with the microglial marker IBA1 (red) or the astrocyte marker GFAP (blue) (c). (d) GFP+ (green) nuclei overlap extensively with native *Sst* mRNA (red). (**B**) Schematic of overall experimental workflow. (1) The dorsal segment from one side of the lumbar spinal cord is dissected. (2) Nuclei are liberated by Dounce homogenization in nuclear lysis buffer. (3) Nuclei are then captured using Fluorescence Assisted Nuclear Sorting. (4) The isolated RNA is processed for RNA-seq. (**C**) GFP+/DAPI+ nuclear were captured from SST^GFP^ (left). No GFP+ nuclei were present in Sun1-GFP^*(fl*/+*)*^ control animals; all DAPI+ nuclei were captured.

In order to specifically tag the nuclei of SST neurons in the dorsal horn, we crossed the *Sst-ires-Cre* mouse line by the Cre-dependent INTACT reporter line, *Sun1-GFP*. We hereafter refer to offspring of this cross as SST^GFP^. In lumbar spinal cord sections from these mice, GFP-labeled nuclei were present in the dorsal horn, with the most abundant distribution in the superficial laminae (I-II) and some sparse expression in the deeper dorsal horn (III-V) (Fig. 1A), consistent with the previously reported expression of SST interneurons in this region^6^.

Combined immunostaining and *in situ* hybridization showed that that GFP + nuclei co-localized extensively with native *Sst* mRNA expression (81.8% ± 2.4%, n = 5), as well as with the neuronal marker NeuN, but not with GFAP or IBA1, markers of astrocytes and microglia, respectively (Fig. 1A). Staining with the nuclear dye DAPI showed nuclear GFP signal localized at the periphery of the DAPI+ nucleus (Fig. 1A, inset).

Having established that the SST^GFP^ mouse faithfully labels the majority of *Sst*-expressing neurons in the dorsal horn, we used Fluorescence Activated Nuclear Sorting (FANS) to selectively collect GFP+ nuclei from the lumbar dorsal segment of 8-week old SST^GFP^ mice (Fig. 1B)^32^. To distinguish nuclei from cellular debris, DAPI was used to stain all intact nuclei. Five thousand (5000) GFP+/DAPI+ nuclei were sorted (n = 3 spinal cords/mice?) directly into lysis buffer. For comparison, the same number of total dorsal horn nuclei (DAPI+/GFP−) was captured from Cre-negative *Sun1-GFP*^*(fl*/+*)*^ control mice (n = 3) (Fig. 1C). Total RNA was isolated from the samples and RNA-seq libraries were generated.

### Transcriptional Profiling of SST neurons

**Figure 2:**
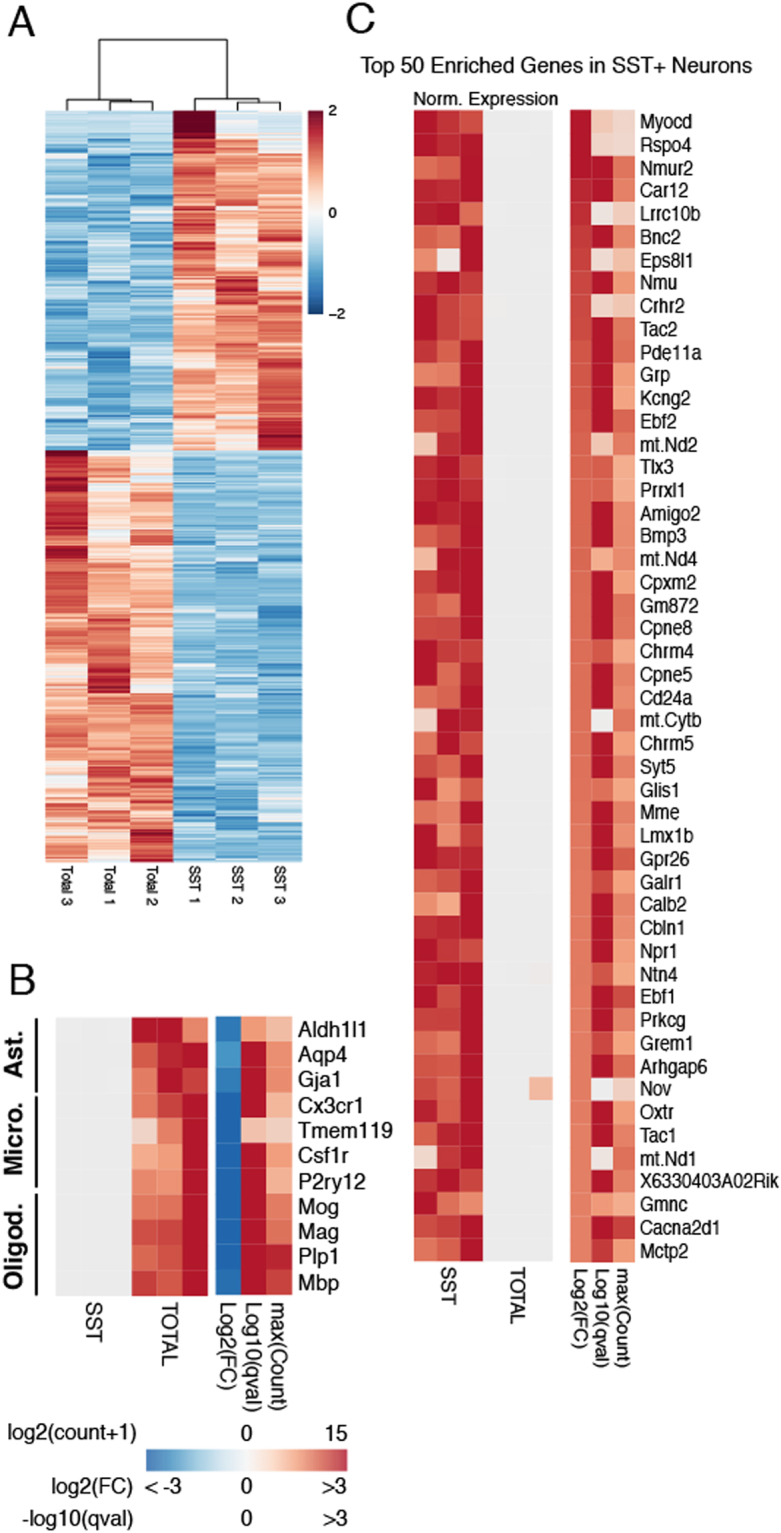
Differential Gene Expression in SST Neurons. (**A**) Hierarchical clustering of SST neurons and total dorsal horn nuclei reveals widely divergent gene expression patterns. Color scale indicates Z-score of normalized expression values. (**B**) Non-neuronal markers are depleted from SST neurons. Ast. = Astrocytes, Micro. = Microglia, Oligod. = Oligodendrocytes. In the left heatmap, the expression level in each sample is normalized by the maximum expression level in the row (Norm. Expression). The right heatmap shows the log2-Fold Change (log2FC), −log10 of the q-value (adjusted p-value) and the log2 of the maximum value of counts for each gene plus a psuedocount of 1 (maxCount). Note: Positive values for log2FC indicate enrichment in SST compared to Total, while negative values indicate enrichment in Total compared to SST. (**C**) Top 50 Differentially Expressed Genes in SST neurons. Genes are ordered by log2FC value from highest to lowest. Criteria for inclusion of genes: log2FC > 1, q-value < 0.01 and CV < 65% and a minimum of 20 counts for all samples.

RNA-seq was performed on the libraries generated from spinal nuclei. The average sequencing depth per sample was 25.8 million reads. We detected 13,342 genes (coding and non-coding) using a normalized count cutoff of 10 (Supplemental Table 1). Pearson correlation hierarchical clustering indicated that the transcriptional profiles of the SST^GFP^ population differed markedly from those of the total population (Fig. 2A). To identify specific genes enriched in SST neurons, we performed differential gene expression analysis, comparing the SST^GFP^ profiles to those of the total nuclei. We observed 901 protein-coding, differentially expressed genes (DEGs) with a log2-fold change (log2FC) > 1, counts > 20, and an adjusted p-value (q-value) < 0.01. SST neurons were greatly depleted of glial markers such as *Aldh1l1*, *Cx3cr1* and *Mog*, representing astrocytes, microglia and oligodendrocytes, respectively (Fig. 2B). Among the top 50 DEGs (Fig. 2C, Table 1), numerous genes with known expression in the dorsal horn and roles in nociception were detected, supporting the validity of both the technique and our results. Examples of known pain and dorsal horn genes include *Nmur2*, *Nmu*, *Tlx3*, *Prrxl1*, *Tac1*, *Lmx1b*, *Prkcg*, *Cacna2d1* and *Grp*^29^. Numerous novel genes without previous description in the dorsal horn also appeared among the top 50 DEGs in SST neurons, several of which we validated and describe below.

**Table 1:** Top 50 differentially expressed genes in SST neurons compared to total dorsal horn cells.

Gene	log2FC	Wald Test Statistic	q-value	log2 (Max Count)
*Myocd*	3.5	5.2	2.2E-06	4.9
*Rspo4*	3.3	4.9	8.1E-06	4.8
*Nmur2*	3.1	10.0	8.4E-22	10.1
*Car12*	2.8	17.3	1.6E-64	9.4
*Lrrc10b*	2.8	4.5	5.6E-05	5.4
*Bnc2*	2.6	14.3	3.0E-44	9.2
*Eps8l1*	2.6	4.8	1.8E-05	6.1
*Nmu*	2.5	12.0	4.2E-31	8.2
*Crhr2*	2.5	4.9	8.6E-06	5.8
*Tac2*	2.5	17.1	7.7E-63	10.0
*Pde11a*	2.4	17.1	2.2E-63	10.4
*Grp*	2.3	8.7	7.8E-17	8.0
*Kcng2*	2.3	9.1	2.6E-18	7.5
*Ebf2*	2.3	17.7	2.2E-67	10.9
*mt-Nd2*	2.2	5.3	1.6E-06	9.7
*Tlx3*	2.2	7.3	4.4E-12	7.0
*Prrxl1*	2.2	7.2	1.1E-11	7.1
*Amigo2*	2.2	14.0	2.5E-42	8.9
*Bmp3*	2.2	12.6	1.5E-34	8.9
*mt-Nd4*	2.2	5.8	9.2E-08	8.9
*Cpxm2*	2.1	9.2	1.9E-18	7.7
*Gm872*	2.1	14.2	1.9E-43	10.1
*Cpne8*	2.1	16.0	2.9E-55	10.0
*Chrm4*	2.1	7.4	2.1E-12	7.3
*Cpne5*	2.1	12.9	5.4E-36	9.4
*Cd24a*	2.1	12.4	3.8E-33	8.9
*mt-Cytb*	2.1	4.3	1.5E-04	10.0
*Chrm5*	2.1	8.4	1.1E-15	7.8
*Syt5*	2.1	13.4	1.0E-38	9.5
*Glis1*	2.0	7.0	4.4E-11	7.4
*Mme*	2.0	10.6	2.1E-24	9.6
*Lmx1b*	2.0	9.5	1.1E-19	8.7
*Gpr26*	2.0	21.8	4.0E-102	11.4
*Galr1*	2.0	7.7	4.2E-13	7.7
*Calb2*	2.0	9.0	9.1E-18	9.3
*Cbln1*	2.0	12.2	4.9E-32	8.8
*Npr1*	2.0	8.9	1.6E-17	7.9
*Ntn4*	2.0	7.5	1.6E-12	7.3
*Ebf1*	2.0	21.7	9.5E-102	12.2
*Prkcg*	1.9	13.1	4.5E-37	9.5
*Grem1*	1.9	7.7	2.6E-13	7.6
*Arhgap6*	1.9	14.7	1.1E-46	10.5
*Nov*	1.9	3.7	1.2E-03	5.2
*Oxtr*	1.9	9.1	2.2E-18	8.0
*Tac1*	1.9	13.2	1.7E-37	9.4
*mt-Nd1*	1.9	4.5	6.1E-05	10.3
*6330403A02Rik*	1.9	13.2	1.9E-37	9.4
*Gmnc*	1.9	6.2	7.4E-09	7.1
*Cacna2d1*	1.9	18.7	3.2E-75	12.8
*Mctp2*	1.9	7.9	5.9E-14	8.0

For each gene we report: gene, log2(fold change), Wald Test Statistic, q-value (adjusted p-value) and the log2(Max Count), which is log base-2 of the maximum normalized count value for each gene. n = 3/group.

### Functional Classes of Genes Expressed by SST neurons

**Figure 3:**
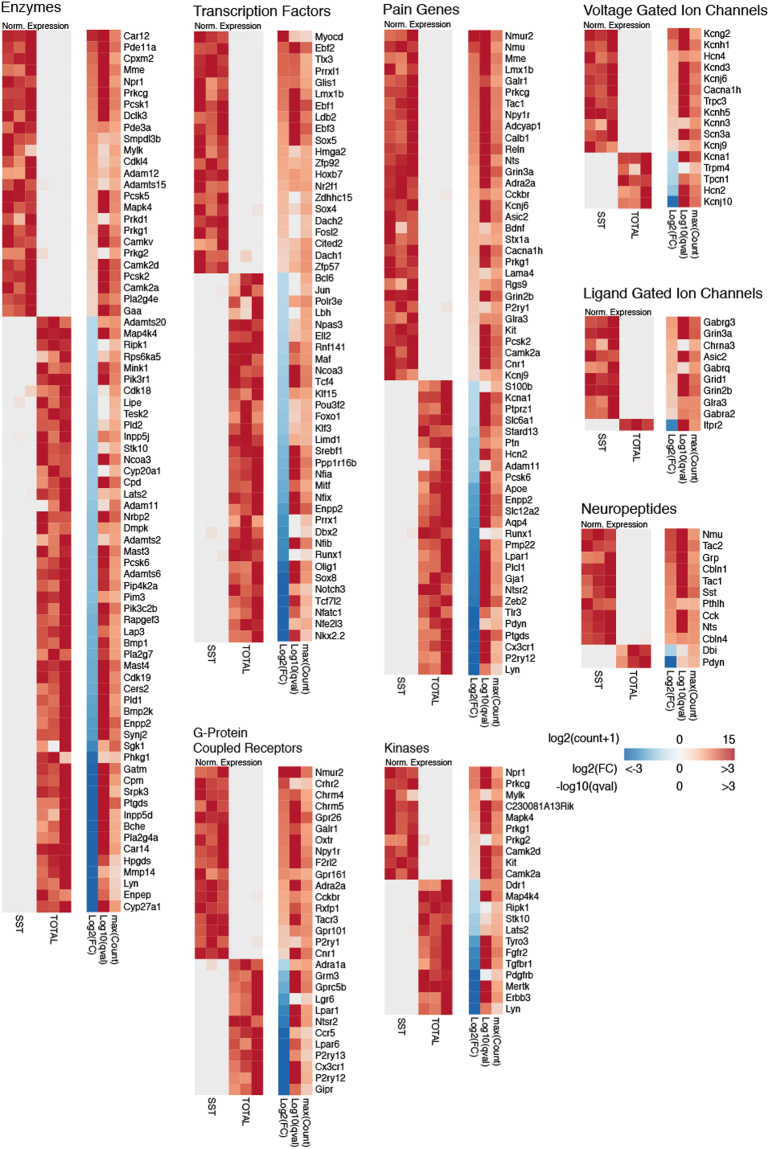
Enrichment of Genes from Selected Functional Classes in SST Neurons. Differential expression of genes in SST neurons compared to total dorsal horn nuclei grouped by functional classes. For each class, in the left heatmap, the expression level in each sample is normalized by the maximum expression level in the row (Norm. Expression). The right heatmap shows the log2-Fold Change (log2FC), −log10 of the q-value (adjusted p-value) and the log2 of the maximum value of counts for each gene plus a psuedocount of 1 (maxCount).

To further examine enrichment of genes that are pharmacologically tractable, we grouped DEGs into relevant functional classes (Fig. 3).

### Enzymes

*Prkcg* encodes for Protein Kinase C gamma isoform (PKCγ), which marks a class of excitatory interneuron involved in mechanical allodynia and contributes to the intracellular signaling cascade that underlies this role^33^. Previous histological studies have shown that PKCγ colocalizes with approximately one-third of SST-expressing interneurons in lamina I-II^34^. Consistently, *Prkcg* is one of the most highly enriched enzymes and is among the top 50 DEGs overall (+1.9 log2FC) in SST neurons.

*Mme* encodes for membrane metallo-endopeptidase, also known as neprilysin, which has been implicated in pain processing via its role in the breakdown of enkephalins and other peptides^35^. *In situ* hybridization studies showed strong and specific expression in the superficial (laminae I-II) dorsal horn of the rat, but more precise characterization of its expression is lacking^36^. Our results here indicate that *Mme* is enriched in SST neurons (+2.0 log2FC).

Within the enzyme class, we also discovered several novel targets with no previously described role in pain or expression in the dorsal horn.

*Car12* encodes for Carbonic Anhydrase 12, which is a membrane spanning carbonic anhydrase whose catalytic domain faces the extracellular space^37^. In the Allen Brain Atlas (ABA) *Car12* mRNA is present in several brain regions, with the highest expression in the striatum and hippocampal formation^38^, and the Human Protein Atlas (HPA) shows CAR12 most abundantly in the skin, bone marrow, kidney, gastrointestinal tract and kidney^37^. A recent large scale single-cell RNA-seq study in the visual cortex identified *Car12* as a unique marker of Layer 6 cortical neuron subtype^39^, but apart from this, virtually nothing is known about the expression and function of *Car12* in the spinal cord.

**Figure 4:**
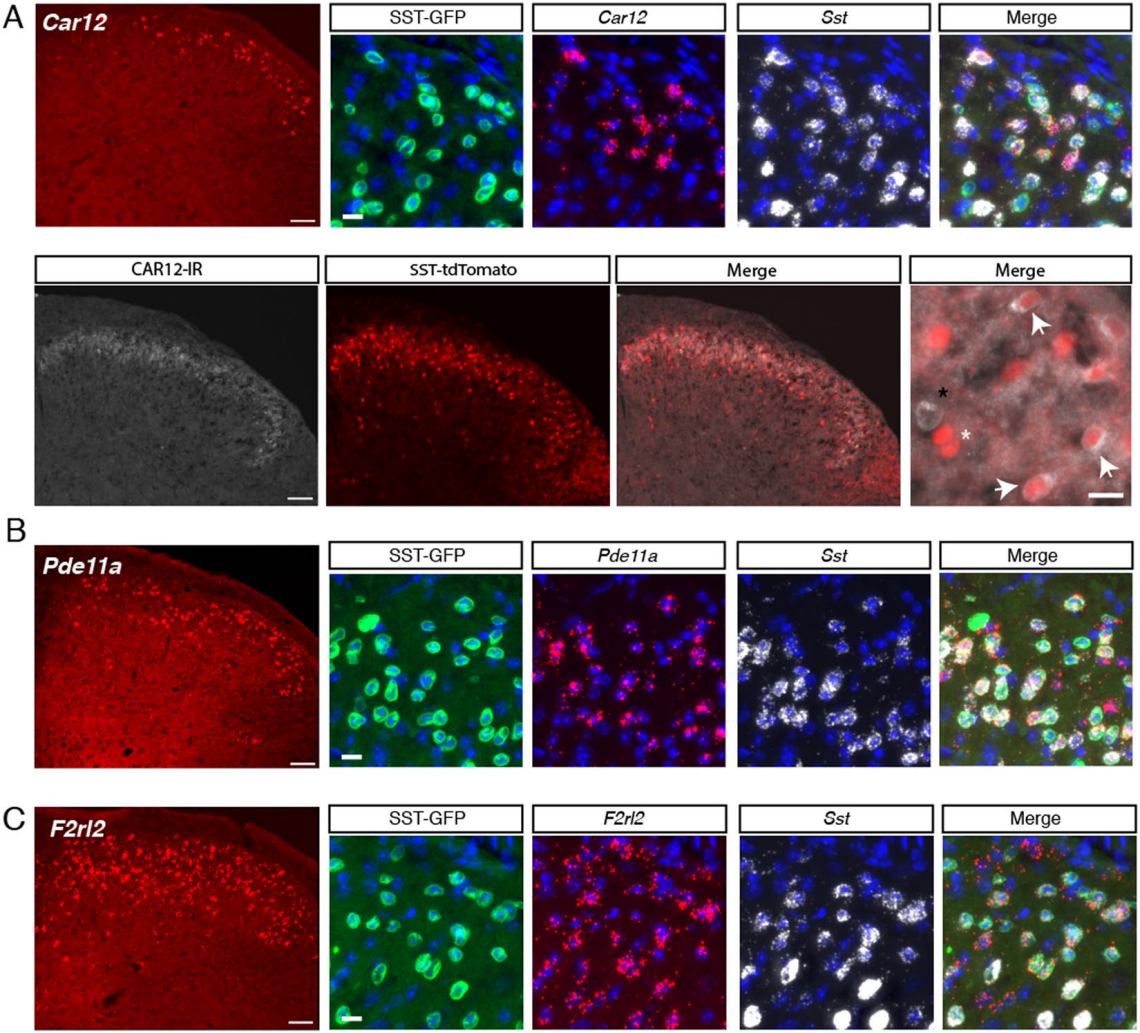
Expression of the novel genes *Car12*, *Pde11a* and *F2rl2* in SST neurons in the dorsal horn. (**A**) *Car12* (red) *in situ* hybridization in the dorsal horn at low magnification (20x). Scale = 50 μm. High magnification (40x) image of GFP, *Car12*, *Sst* and merge. DAPI (blue) in images. Scale = 10 μm. Immunohistochemistry of CAR12-IR, (g) SST-Tomato and (h) merge at magnification (20x). Scale = 50 μm. High magnification (40x) image of SST-Tomato and CAR12-IR. Scale = 10 μm. White arrows indicate cells with tdTomato and CAR12-IR co-localization. White asterisk indicates tdTomato+ cells that lack CAR12-IR and black asterisk indicates a CAR12-IR+ without tdTomato. (**B**) *Pde11a* (red) *in situ* hybridization in the dorsal horn at low magnification (20x). Scale = 50 μm. High magnification (40x) image of GFP, *Pde11a*, *Sst* and merge. DAPI (blue) in images. Scale = 10 μm. (**C**) *F2rl2* (red) *in situ* hybridization in the dorsal horn at low magnification (20x). Scale = 50 μm. High magnification (40x) image of GFP, *F2rl2*, *Sst* and merge. DAPI (blue) in images. Scale = 10 μm.

To validate the expression of *Car12*, we performed both *in situ* hybridization and immunostaining. *In situ* hybridization showed strong expression of *Car12* transcripts exclusively in the superficial dorsal horn that co-localized extensively with *Sst* transcript as well as GFP + nuclei (Fig. 4A and Table 2). Nearly half of all cells expressing *Sst* transcript expressed *Car12* (46.9% ± 0.7%, n = 5), and the majority of *Car12*-expressing neurons expressed *Sst* (69.2% ± 0.1%, n = 5). 42.7% ± 2.1% of GFP+ neurons co-expressed both *Car12* and *Sst* mRNA. Because *Car12* is an extracellular carbonic anhydrase (ECA), it is expected to localize to the plasma membrane of the cells in which it is expressed. To better visualize the somata of SST neurons, we employed an SST-Tomato mouse, which expresses cell-filling tdTomato^6^. Immunostaining with an antibody against mouse CAR12 showed CAR12-immunoreactivity (CAR12-IR) localized on the membrane of SST-Tomato neurons in superficial laminae, consistent with the findings for *Car12*-mRNA Fig. 4A) and its known function as an extracellular-facing enzyme.

**Table 2:** Quantification of Gene Co-Expression by *In Situ* Hybridization.

*Pde11a*		*Car12*		*F2rl2*	
*Pde11a*+/*Sst*+	50.8% (1.3)	*Car12*+/*Sst*+	46.9% (0.7)	*F2rl2*+/*Sst*+	59.6% (2.0)
*Sst*+/*Pde11a*+	64.2% (1.9)	*Sst*+/*Car12*+	69.2% (0.1)	*Sst*+/*F2rl2*+	57.1% (0.8)

For *Car12, Pde11a and F2rl2*, the percentage co-expression with *Sst* is indicated. Standard error of the mean is in parentheses. n = 3–5.

Phosphodiesterase 11A (PDE11A) is a dual specificity phosphodiesterase that catalyzes the breakdown of the cyclic nucleotides cyclic adenosine monophosphate (cAMP) and cylic guanosine monophosate (cGMP). PDE11A exhibits restricted expression in the brain of mouse, predominating in the ventral hippocampus^40^ Sunkin:2012bj}. Protein analysis of bulk tissue from mouse spinal cord and dorsal root ganglia (DRG) detected PDE11A but did not specify the cell type^41^. In humans, *PDE11A* mRNA is found most abundantly in the prostate and spinal cord according to the GTEx database^42^. Knockout studies of PDE11A have implicated this gene in regulating social behaviors and anxiety^43^. In the dorsal horn, cyclic nucleotide signaling contributes to pain-related neuronal plasticity, and other members of the phosphodiesterase family have been directly implicated in the pathogenesis of inflammatory and neuropathic pain^44^. Accordingly, we selected *Pde11a* for further examination. *In situ* hybridization of *Pde11a* transcript revealed a strong and specific signal that predominated in the superficial laminae (Fig. 4B and Table 2). Half of all *Sst*-expressing neurons co-expressed *Pde11a* (50.8% ± 1.3, n = 3), and conversely, nearly two-thirds of *Pde11a*-expressing neurons co-expressed *Sst* mRNA (64.2% ± 1.9%, n = 3). More than half of all GFP+ nuclei in the dorsal horn co-localized with both *Pde11a* and *Sst* mRNA (55.8% ± 3.0%, n = 3).

### Transcription Factors

Transcription factors are responsible for the specification of neuronal identity^45^. Much work has been conducted on the role of specific transcription factors in the development of dorsal horn neurons involved in pain and itch^46^. Nearly all of the well-studied transcription factors that specify excitatory interneuron development were enriched in SST neurons, including *Tlx3*, *Lmx1b*, *Prrxl1*, *Sox5*, and *Ebf1-3*^47,48^, in support of the biological accuracy of our RNA-seq results. Enriched transcription factors without known functions in dorsal horn interneurons included *Myocd* (+3.5 log2FC), *Glis1* (+2.0 log2FC) and *Ldb2* (+1.7 log2FC)^48^. In addition, a zinc-finger-containing protein, Basonuclin 2 (*Bnc2*) was among the most highly enriched genes overall. While not a classical transcription factor, by virtue of its zinc finger domain and nuclear localization signal, *Bnc2* regulates transcriptional events^49^. A recent report that used a LacZ reporter mouse demonstrated that in the central nervous system, *Bnc2* is only expressed in a small subset of dorsal horn cells^49^, which is consistent with our transcriptomic results.

### G-Protein Coupled Receptors

G-protein coupled receptors (GPCRs) are one of the most important classes of drug targets, making up nearly half of all human drug targets^50^. GPCRs play diverse roles in neurons, functioning prominently as neurotransmitter and neuromodulator receptors^51^. Numerous GPCRs that have been implicated in pain processing in the spinal cord were significantly enriched in the SST population, including *Nmur2*, *Galr1*, *Npy1r*, and *Cckbr*. In support of our transcriptomic results, previous reports have shown that *Galr1* and *Npy1r* are preferentially expressed in excitatory dorsal horn neurons under the control of *Tlx3*^52^. Interestingly, two members of the muscarinic acetylcholine receptor family, *Chrm4* (+2.1 log2FC) and *Chrm5* (+2.1 log2FC), were also significantly enriched.

*Nmur2* is one of two G-protein coupled receptors that bind to the pleotropic neuropeptide neuromedin (NMU). Knockout studies of NMUR2 demonstrated that this receptor contributes to nociception^53^. Injection of intrathecal NMU enhances synaptic transmission as measured by electrophysiological studies, and produces mechanical and thermal hyperalgesia in behavioral assays^54,55^. Apart from its presence in the dorsal horn, further characterization of the neurons that express *Nmur2* has not been performed. As the third most enriched gene in our top 50 DEGs (Fig. 2C), *Nmur2* is one of the more distinctive genes observed, suggesting that it is uniquely expressed by SST neurons. Using *in situ hybridization*, we found *Nmur2* to be expressed nearly exclusively in SST neurons (Figure S1A). As with other genes we examined, only a portion of the *Sst*-expressing neurons co-express *Nmur2*, indicating that these neurons are a unique subpopulation within the broader SST neurons.

We also identified several novel GPCRs whose presence in the dorsal horn has not been described.

*F2rl2* encodes for the protein PAR3, which is a G-protein coupled receptor in the family of Protease Activated Receptors (PARs), of which there are four (PAR1-4)^56^. As their names suggest, PARs are activated when proteases such as thrombin or trypsin cleave a portion of the receptor, creating a tethered ligand that then acts as an intramolecular agonist for the receptor. PAR1, 2 and 4 have all been implicated in pain, with PAR2 being the most well-studied^57–59^. PAR3’s function, however, remains elusive, with neither the ligand nor mode of action of this receptor known. *PAR3* mRNA in humans is present in the brain, particularly the hypothalamus, and outside the CNS it is found most abundantly in gastrointestinal tissues according to the GTEx database^42^. In light of the role of the other PARs in pain and itch, the enrichment of PAR3 in SST neurons is suggestive. Thus, we confirmed the expression of *Par3* miRNA by *in situ* hybridization (Fig. 4C and Table 2). *F2rl2* mRNA strongly expresses in the superficial dorsal horn and co-localizes with the majority of *Sst-*expressing neurons in the dorsal horn (59.6% +/− 2.0%, n = 5). More than half of all *F2rl2*-expressing neurons also express *Sst* mRNA (57.1% +/− 0.8%, n = 5). Future functional studies using knockout mice or pharmacological tools will be needed to determine the role of this gene in spinal nociception.

*Gpr26* is an orphan G-protein coupled receptor that is coupled to G_s_ and for which an endogenous ligand is not known^60^. Studies with *Gpr26* knockout mice have implicated this receptor in anxiety, depression-like behaviors and obesity, but no known role in pain is known^61^. As one of the most enriched GPCRs (+1.9 log2FC) and one of top 50 DEGs (Fig. 2C), we examined the expression of *Gpr26* using *in situ* hybridization. We found this receptor broadly expressed in the dorsal horn, and co-localized with a subset of SST neurons (Figure S1B).

### Kinases

Kinases play key roles in intracellular signaling cascades and are frequent targets for pharmacological modulation. Several kinases were enriched in the SST population. *Prkcg*, which has a known role in pain, was the most highly enriched and most distinctive. Other moderately enriched kinases include members of the Calcium-calmodulin Kinase family (*Camk2a* and *Camk2b*), cyclic-GMP-dependent kinase family (*Prkg1* and *Prkg2*) and Mitogen Activated Protein Kinase family (*Mapk4*).

### Ion Channels

Our RNA-seq results uncovered several previously described ion channels that express in the dorsal horn. *Trpc3* is a TRP channel family member that has been shown to be enriched in the dorsal horn, but no further investigation has characterized its localization or function in spinal neurons^48^. *Hcn4*, which is a hyperpolarization-activated cyclic nucleotide-gated channel, has been shown to co-localize with PKCγ interneurons in lamina IIi, some of which are *Sst*-expressing^62^. *Cacna2d1*, which is the target of the clinically effective gabapentinoids, was also highly enriched in SST neurons and with nearly the highest normalized counts of all genes analyzed (Table 1). Many studies of *Cacna2ad1* in pain have examined presynaptic mechanisms in sensory afferents^63^, but the high expression of this channel subunit in SST neurons suggests a role in these spinal neurons as well. Several ligand-gated ion channels were also enriched, including *Grin3a*, which is an NMDA-receptor subunit known to express in excitatory dorsal horn neurons^52^ and the acid-sensing *Asic2* channel, which has also been described in dorsal horn neurons^64^.

Among previously uncharacterized ion channels, the *Kcng2* channel was the most highly enriched in SST neurons. *Kcng2* encodes a gamma subunit of the Kv6 family of voltage-gated potassium channels (Kv6.2). Little is known about the functional role of *Kcng2*. It is highly expressed in myocardial tissue, and so a role in cardiac function has been proposed^65^, but no study of *Kcng2* has been conducted in the nervous system. *Kcng2* is enriched in SST neurons (+2.3 log2FC) compared to all cells in the dorsal horn.

#### Neuropeptides

Neuropeptides play key roles as neuromodulators and often define different populations of neurons, such as is the case with *Sst*. We found several neuropeptides to be enriched in SST neurons. Many of these neuropeptides have been identified and investigated in the dorsal horn, including *Tac2*, *Grp* and *Sst*^66,67^. As expected, the defining neuropeptide, *Sst* is enriched (+1.8 log2FC). Also, in further support of our methodology, the neuropeptide *Pdyn*, which is exclusively expressed by inhibitory dorsal horn neurons^68^, was strongly depleted in SST neurons (−3.8 log2FC).

The neuropeptide Neuromedin U (NMU) is encoded by the *Nmu* gene and exerts wide-ranging effects both in the periphery and CNS. NMU is abundant in the spinal cord and knockout of *Nmu* in the mouse produces large deficits in the hot plate and formalin tests^54^. Exogenous NMU enhances nociceptive behaviors, including mechanical sensibility^55^. The cellular expression profile of *Nmu* in the spinal cord is unknown. Our RNA-seq data indicate that *Nmu* is enriched (+2.5 log2FC) in SST neurons, suggesting that some SST neurons use this neuropeptide in nociceptive signaling. As we demonstrated, the receptor for NMU, *Nmur2*, was also highly enriched in a subset of *Sst*-expressing neurons in the superficial dorsal horn (Figure S1A).

*Tac2* encodes the neuropeptide Neurokinin B^34^. *Tac2* was highly enriched in the SST population. This neuropeptide has been identified in dorsal horn neurons and found to overlap in part with PKCγ-expressing neurons, some of which are also *Sst*-expressing^67^. However, toxin-mediated ablation of the *Tac2*-lineage neurons in the spinal cord did not affect mechanical pain behaviors^6^, in contrast to the dramatic effect of ablation of the SST neurons. This indicates that the subset of SST neurons that expresses *Tac2* is dispensable for mechanical pain behaviors. What role these *Tac2*/*Sst* co-expressing neurons plays in pain remains to be determined.

*Grp* encodes the Gastrin Related Peptide (GRP), a neuropeptide that has been well-studied for its role in itch transmission in the dorsal horn^69,70^. Recent work has demonstrated that GRP is expressed by a subset of dorsal horn interneurons and is used for pruriceptive transmission. Moreover, it is known that these GRP-expressing interneurons co-express the Natriuretic Peptide Receptor A (*Npr1*) and release GRP when NPR1 is activated by its cognate ligand Natriuretic Polypeptide B (*Nppb*)^69^. Our RNA-seq data indicate that both *Grp* (+2.5 log2FC) and *Npr1* (+2.0 log2FC) are enriched in SST neurons, suggesting that the pruriceptive *Grp*+/*Npr1*+ population is subsumed in the SST population. Consistently, a recent study demonstrated that optogenetic activation of SST neurons in the dorsal horn elicited strong light-induced itch behaviors^71^. Another study used a chemogenetic approach to manipulate *Grp*-expressing spinal neurons and found that this population mediates both pain and itch in an intensity-dependent manner^72^.

### Additional Genes

Other genes with high enrichment and relatively high counts appeared that do not fall into the functional classes described above (Table 1). *Bmp3* encodes Bone Morphogenetic Protein 3, which is a secreted ligand of the TGF-β family. *Bmp2, Bmp4* and *Bmp7* play critical roles in the specification of dorsal spinal neurons, but no role for *Bmp3* has been described, although the expression of BMP3 in the adult dorsal horn by immunohistochemistry has recently been described^73^. As a secreted ligand, BMP3 may function in intercellular signaling in the dorsal horn.

C*pne5* and *Cpne8* encode for Copine 5 and Copine 8, respectively. Copines are a family of calcium-dependent phospholipid-binding proteins. Copine 6 has been shown to regulate excitatory synapses in the hippocampus by translating calcium signals into changes in dendritic spine structure through Rac1 recruitment^74^. Copine 5 and Copine 8 may function analogously in regulating the excitatory synapses of SST neurons in the dorsal horn and warrant further study.

#### Pathway Analysis

**Figure 5:**
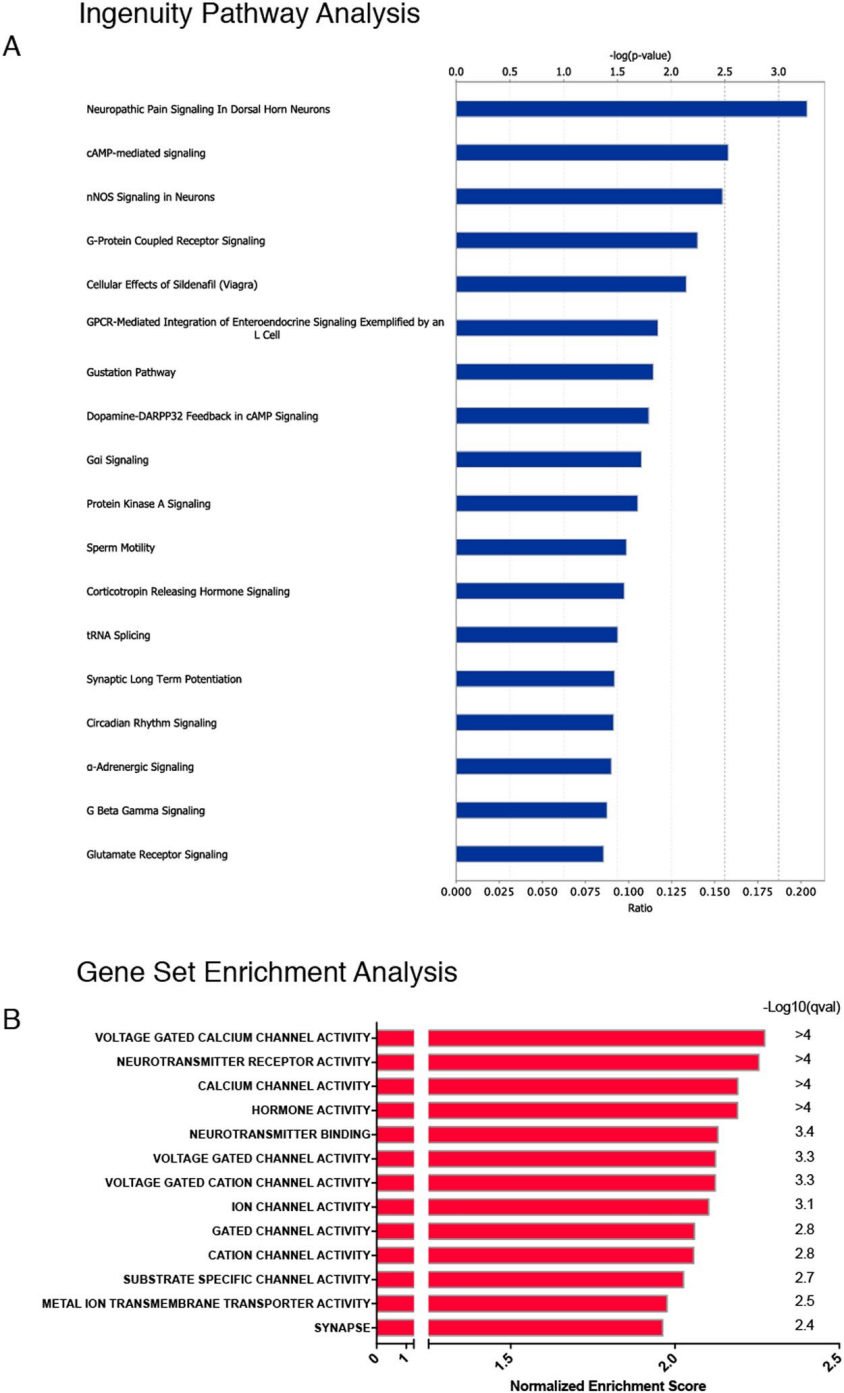
Pathway analysis and Gene Set Enrichment Analysis of SST neurons. (**A**) Ingenuity Pathway Analysis reveals multiple enriched pathways in SST neurons. Top axis = −log(p-value). Bottom axis = ratio of enrichment in canonical pathway. Data were analyzed through the use of IPA (QIAGEN Inc., https://www.qiagenbioinformatics.com/products/ingenuitypathway-analysis). (**B**) Gene Set Enrichment Analysis of SST neurons.

Genes that participate in functional pathways are often coordinately expressed. To determine whether known signaling pathways are enriched in SST neurons, we applied Ingenuity Pathway Analysis. Using the same selection criteria as for single genes (q < 0.01, log2FC > 1), we identified several enriched pathways and ranked them by their statistical significance (Fig. 5). Top among the pathways was “Neuropathic Pain in Dorsal Horn Neurons”, which is consistent with the known role of SST neurons in neuropathic pain models. The next identified pathway was cAMP-mediated signaling followed by “nNOS signaling in neurons” and “G-Protein Coupled Receptor signaling”. To complement the Ingenuity analysis, we also applied Gene Set Enrichment Analysis (GSEA), which is used to identify coordinate changes in the expression of groups of functionally related genes^27^. From this analysis, the top gene set was Voltage Gated Calcium Channel Activity, followed by Neurotransmitter Receptor Activity and Calcium Channel Activity. These gene set enrichments suggest that calcium signaling is particularly important in the SST neurons.

## Discussion

In this study we present a comprehensive transcriptomic profile of SST neurons in the spinal dorsal horn of the mouse and identify several novel differentially expressed genes that could be attractive therapeutic targets and population markers.

We optimized the INTACT method for spinal tissue in order to profile the transcriptome of SST neurons in the dorsal horn. To our knowledge, this is the first demonstration of cell type-specific transcriptomic profiling of a neuron population from this region, and the first to use nuclei in lieu of cells. Our results show that neuronal nuclei are suitable surrogates for whole cells for RNA-seq profiling of neuronal populations of the dorsal horn, and thus this work represents an important technical advance that will facilitate future interrogation of specific dorsal horn cell populations in normal and pathological conditions (e.g. inflammatory or neuropathic models). Previous gene expression profiling studies have looked at bulk RNA from spinal cord tissue in such pathological pain models, and the resulting signatures are largely dominated by immune and glial signal^9^. The spinal cord-adapted INTACT method we present here will allow researchers to better understand the transcriptional plasticity of specific cell populations, especially smaller populations whose signal is obscured by the averaging effects of bulk RNA-seq. Indeed, the novel genes we identified in this study have gone unnoticed in previous genome-wide expression studies^48^ perhaps because they comprise only a small fraction of transcripts in bulk RNA from the spinal cord. By isolating a specific population from all surrounding cells, the signal of such population-enriched genes is unmasked. Moreover, because nuclei carry both DNA and RNA, other genomic applications such as ATAC-Seq, ChIP-Seq or bisulfite sequencing could be profitably applied in tandem with RNA-seq to elucidate pain-related epigenetic plasticity in a cell type-specific manner, which is increasingly appreciated to play key roles in pathological pain^75,76^.

Transcriptomic profiling of SST neurons with INTACT allowed us to identify numerous genes that are enriched in these neurons in the dorsal horn. Some of these enriched genes, such as *Tlx3*, *Lmx1b*, *Pkcg*, *Tac2*, *Grp*, *Calb2, Ebf1-3*, and *Nmur2* were already known to express preferentially in excitatory neurons of the dorsal horn, of which the SST population comprises a large proportion^34^. Our results overlap to a high degree with the catalog of dorsally enriched genes identified through microarray screening by Li *et al*.^48^. Identification of these known targets lends support for the overall biological validity of our findings.

The major aim of this work was to discover novel genes that are enriched in SST neurons. Indeed, we report here several genes whose expression in the dorsal horn was previously unknown and whose annotated functions make them attractive for pharmacological intervention. Using *in situ* hybridization, we characterized the expression of three novel genes that were highly enriched in SST neurons: *Pde11a*, *Car12*, *F2rl2*. Our results show that each of these genes is expressed in a subset of *Sst*-expressing neurons in the superficial dorsal horn, with *Pde11a* and *Car12* showing particularly restricted expression in laminae I-II. Further studies using specific genetic and pharmacological tools will be needed to determine whether and how each gene contributes to pain processing in *Sst*-expressing neurons.

By what mechanisms might these genes contribute to pain processing in SST and other spinal neurons? As a dual-specificity phosphodiesterase, PDE11A has the ability to modulate intracellular cAMP and cGMP levels by catalyzing their breakdown. Several studies have found that cAMP is increased in the dorsal horn in the setting of inflammation or injury and that inhibition of the cAMP pathway relieves pain hypersensitivity^77–79^. The contribution of cGMP is equivocal, with some studies show pro-nociceptive and others showing anti-nociceptive effects^72^. Thus, PDE11A could either enhance or mitigate pain depending on context and the net activity of other phosphodiesterases.

Although CAR12 has not been studied in the CNS, other extracellular carbonic anhydrases (ECAs) such as CAR4 and CAR14 have been well-studied in the brain, where they have been shown to modulate neuronal activity by regulating extracellular pH^74,80^. In the hippocampus, several studies have shown that ECAs regulate excitatory synaptic transmission via pH-mediated modulation of NMDA receptor activation^81^. Given that the majority of the SST neurons in the dorsal horn are excitatory, the distinct enrichment of *Car12* in these neurons may indicate that it regulates NMDA-mediated excitatory synaptic transmission in a manner analogous to that observed in the hippocampus. Accordingly, activation of CAR12 could inhibit pain by attenuating NMDA activity in SST+ excitatory neurons.

F2RL2 is a GPCR coupled to the alpha subunit G_q_, suggesting that this receptor may modulate neuronal excitability via intracellular calcium signaling. Because it is enriched in SST neurons in the superficial dorsal horn, it is conceivable that F2RL2 enhances the excitability of this population, and thus would be pro-nociceptive. But several questions remain. What is the endogenous ligand of F2RL2? Does F2RL2 act independently or does it require another PAR as a co-receptor? And is F2RL2 activation alone sufficient to induce SST neuron firing or rather does it modulate the action of glutamatergic excitatory transmission? The development of specific tools to manipulate F2RL2 will enable answers to these questions.

Our findings highlight the transcriptional heterogeneity of the SST population and suggest possible subpopulations. Among the most highly enriched genes are markers for multiple neurochemically distinct excitatory neuron populations. In a quantitative study of lamina I-III excitatory interneurons, Gutierrez-Mecinas *et al*. demonstrated that the SST population subsumes three largely non-overlapping excitatory populations defined by *Grp, Tac2* and *Nts*, leading the authors to conclude that SST broadly marks most excitatory interneurons of the superficial dorsal horn^34^. Our RNA-seq findings are entirely consistent with this study from Gutierrez-Mecinas and support the conclusion that the SST population is comprised of multiple excitatory subpopulations. The restricted expression of *Car12* and *Pde11a* suggests that these genes define subpopulations of excitatory interneurons that may be functionally and neurochemically distinct from other known subtypes. *Nmur2* also appears in a sparse and restricted population that may be distinct as well. Cre driver lines under the control of these genes would be useful to explore the functional role of these populations. In contrast, *F2rl2* has broader expression, and likely marks most or all excitatory interneurons lamina I-III. Accordingly, this GPCR would be better suited as modulator of excitatory synaptic transmission rather than an specific marker.

In addition to looking at single genes we also applied pathway analysis to uncover pathways and functional groups of genes enriched in SST neurons. Using Ingenuity Pathway Analysis, we revealed that SST neurons exhibit enrichment in several druggable pathways including cAMP-, nNOS- and GPCR-mediated pathways. Gene Set Enrichment Analysis revealed an enrichment in calcium channel-related gene sets, pointing toward calcium signaling as an important contributor to the functional properties of SST neurons. Future work that manipulates these pathways as a whole will be needed to understand their contributions to the functional properties of SST neurons.

Our results also highlight some of the limitations of cell type-specific profiling using INTACT. While RNA-seq profiles from nuclei and cells have been shown to be highly concordant, significant differences do exist^18,19,82^. Specifically, genes related to mitochondrial respiration are enriched in whole cells compared to nuclei, while non-coding RNA and genes related to regulation of transcription and RNA metabolic processes are enriched in nuclei^18,19^. Moreover, the agreement between cell and nuclear RNA depends on exonic gene length, which can diminish the ability of nuclear RNA-seq to detect shorter transcripts^19^. These differences should be considered when interpreting nuclear RNA-seq findings. Another limitation is that INTACT and related cell type-specific methods rely on a single Cre driver line to tag a cell population, the specificity of the readout depends on the specificity of the Cre driver line. As we and others have demonstrated, the SST population is internally heterogeneous^6,34^. Intersectional methods that use two recombinases (i.e. Cre and Flp) driven by separate genes could be used with INTACT to more precisely access the transcriptomic signatures of discrete dorsal horn neuronal subtypes^6,83^, but a new intersectional INTACT mouse or viral vector would need to be generated. Lastly, when differential expression analysis is the principal goal, as it was in this current study, consideration must be given to experimental design and the choice of appropriate comparators. In this study, we elected to compare the gene expression from GFP-tagged SST neurons to that of total spinal nuclei from separate animals in order to examine enrichment of genes in the SST population compared to the total pool of RNA in spinal cells. We chose this design in order to minimize the potential for detecting genes that are broadly enriched in neurons compared to glia, rather than specifically enriched in SST neurons, as might have occurred if we used a depleted population (i.e. GFP−/DAPI+) as the comparison. Accordingly, our results likely underestimate the true magnitude of enrichment of genes in SST neurons and still cannot completely circumvent the possibility that some genes identified in this study are markers of neurons as a whole and not specifically SST neurons. An additional experimental caveat is that use of different strains for comparison may have influenced the differential gene expression results, since *Sst-ires*-*Cre* has been shown to affect the expression of some genes^84^. In future studies, more specific comparisons could be made if, instead of using total spinal nuclei for comparison, other Cre lines are used that mark specific neuronal populations (e.g. *Vgat, Vglut2, Nts, Pdyn, Penk*, etc.). These considerations aside, INTACT is a powerful and enabling technology for cell type-specific genomics in the spinal cord.

This study also demonstrates the general utility of nuclei as surrogates for cells in the study of spinal cord transcriptomics. Single-cell genomics is revolutionizing our understanding of cell types in the nervous system^85–87^ and large international efforts are now underway to comprehensively catalog the cells of the mouse and human brain^88^. The spinal cord also stands to benefit greatly from these efforts so that a comprehensive census of the neuronal and non-neuronal cell types in the spinal cord can be determined. Indeed, single-nucleus RNA-seq (snRNA-seq) has emerged as an effective strategy in the profiling of single cells in the brain and spinal cord^85,89^ already. Going forward, we propose that single-nucleus RNA-seq and population-level INTACT be used in a complementary manner. Massively parallel snRNA-seq with high numbers of nuclei would be the most effective way to identify and classify cell types in the spinal cord, but would most likely lack the depth to reveal disease or treatment effects on low- to medium-abundance transcripts^85^. Population-level INTACT and deep sequencing, as we present here, would have the ability to detect transcriptional perturbations from manipulations (e.g. injury, inflammation, drugs) that would be missed by single nuclei.

In summary, our work provides a framework for combining knowledge of functional pain circuitry with transcriptomics to uncover novel genes and to refine our understanding of cell types in the spinal cord. In particular, SST neurons show plasticity under inflammatory and neuropathic pain conditions and respond to inflammatory cytokines and chemokines such as TNF-α and CCL2^90,91^. Thus, specific targeting of signaling transduction in these neurons may lead to the development of new therapeutics for the management of pathological pain, meanwhile leaving physiological pain intact.

## Electronic supplementary material

Supplementary Information

Dataset 1
