# Supplementary material for: Transcriptional Profiling of Somatostatin Interneurons in the Spinal Dorsal Horn
Source: Sci Rep. 2018 May 1;8:6809. doi: 10.1038/s41598-018-25110-7 (PMC5931607; doi:10.1038/s41598-018-25110-7)
Supplement: Supplementary file 1 — Supplementary Information [file 41598_2018_25110_MOESM1_ESM.pdf]

Supplementary Information

Transcriptional Profiling of Somatostatin Interneurons in the Spinal Dorsal Horn

Alexander Chamessian[1, 3, 4], Michael Young[2], Yawar Qadri[1], Temugin Berta[5], Ru-Rong Ji[1,2] , and Thomas Van De Ven[1]

[1]Department of Anesthesiology, Duke University Medical Center, Durham, North Carolina, 27710, USA

[2]Department of Neurobiology, Duke University Medical Center, Durham, North Carolina, 27710, USA

[3]Medical Scientist Training Program, Duke University School of Medicine, North Carolina, 27710, USA

[4]Department of Pharmacology and Cancer Biology, Duke University Medical Center, Durham, North Carolina, 27710, USA

[5]Pain Research Center, Department of Anesthesiology, University of Cincinnati Medical Center, Cincinnati, Ohio, 45267, USA

Supplementary Figure 1

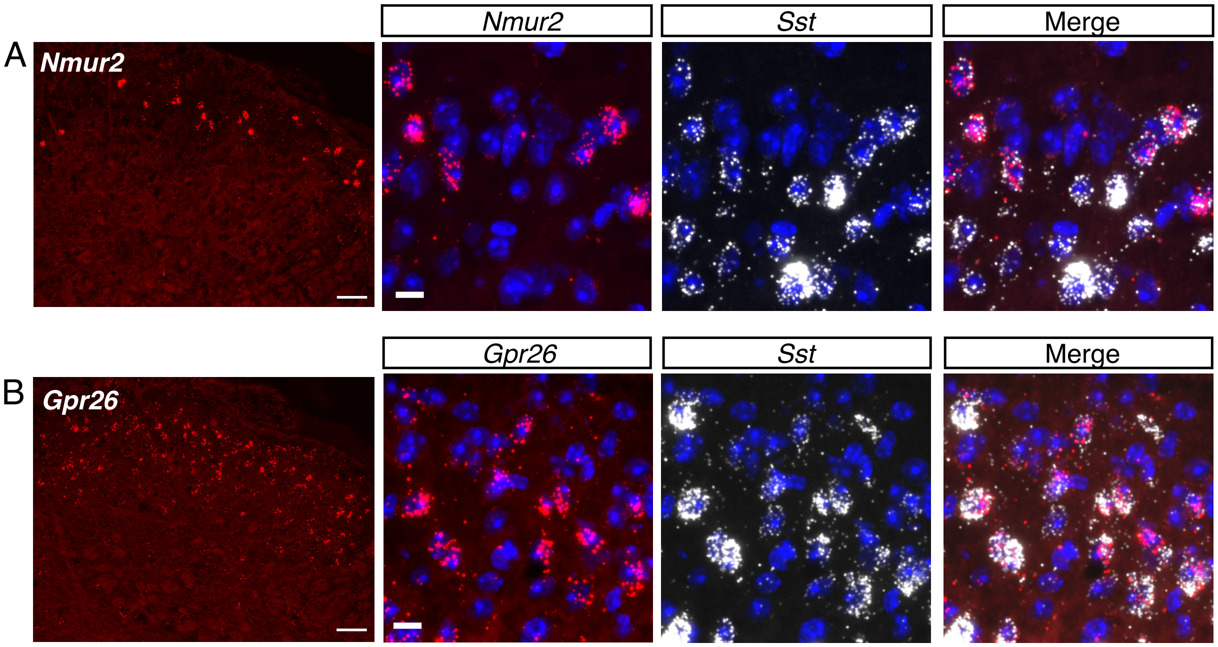

Supplemental Figure 1 - Expression of the G-protein Coupled Receptors *Nmur2* and *Gpr26* in the dorsal horn. (A) *Nmur2* (red) *in situ* hybridization in the dorsal horn at low magnification (20x). Scale = 50 µm. High magnification (40x) image of *Nmur2*, *Sst* and merge. DAPI (blue) in images. Scale = 10 µm. (B) *Gpr26* (red) *in situ* hybridization in the dorsal horn at low magnification (20x). Scale = 50 µm. High magnification (40x) image of *Gpr26*, *Sst* and merge. DAPI (blue) in images. Scale = 10 µm.
